# Microgel Modified UV-Cured Methacrylic-Silica Hybrid: Synthesis and Characterization

**DOI:** 10.3390/ma6093805

**Published:** 2013-09-06

**Authors:** Carola Esposito Corcione, Raffaella Striani, Mariaenrica Frigione

**Affiliations:** Department of Engineering for Innovation, University of Salento, 73100 Lecce, Italy; E-Mails: raffaella.striani@unisalento.it (R.S.); mariaenrica.frigione@unisalento.it (M.F.)

**Keywords:** organic-inorganic hybrids, photopolymerizable acrylic resins, co-continuous nanophases, dual-curing

## Abstract

An innovative photopolymerizable microgel modified UV-cured acrylic-silica hybrid formulation was developed and characterized for possible use as protective coating for different substrates. A deep investigation, aiming at providing a strong scientific basis for the production of organic-inorganic (O-I) hybrids exhibiting phase co-continuity, was firstly carried out. The O-I hybrid first proposed in this study was obtained from organic precursors with a high siloxane content, which are mixed with tetraethoxysilane (TEOS) in such a way to produce co-continuous silica nanodomains dispersed within the crosslinked organic phase, as a result of the sol-gel process. The first part of the research deals with the selection and optimization of suitable systems through appropriate chemical modifications, in order to ensure that curing reactions can be carried out at room temperature and in the presence of UV radiation. Firstly, the silica domains are formed, followed by crosslinking reactions of the acrylic groups in the oligomer via a free radical polymerization. The crosslinking reaction was controlled with the use of a suitable photoinitiator. Most of the experimental work was devoted to understanding the morphology of the hybrid system, both in uncured and cured states, and to assess its final thermal and optical properties, using different experiential techniques.

## 1. Introduction

In recent years, there has been considerable research interest in a new class of materials, generally known as organic-inorganic (O-I) hybrids. These are developed from the concept of intermingling on a nanometric scale the domains of organic (either as linear or network components) and inorganic components, so that, at this scale, the resulting material assumes a character that is a “hybrid” between the two chemical features. The method used for the production of O-I hybrids is effective to obtain interconnected phases with dimensions in the region of five to 50 nm, giving properties that are substantially different from those obtained by the incorporation of discrete particulate or fibrous fillers, even when these have dimensions in the nanometric range, referred to as nanocomposites. The co-continuity of phases, strongly chemically linked to each other, achievable in the production of O-I hybrids, makes it possible to exploit, in the most efficient manner, the properties of the two components [1,2,3,4].

The successful methodology for the production of O-I hybrids has been developed from the sol-gel technology used for the production of ceramic, for instance, employed as coatings. The sol-gel method involves a series of hydrolysis and condensation reactions, starting from a hydrolysable multifunctional alkoxysilane as the precursor for the inorganic domain formation [5,6]. The use of a suitable coupling agent allows one to obtain a strictly interconnected network, preventing macroscopic phase separation. The coupling agent provides bonding between the organic and the inorganic phases, thus giving rise to well-dispersed nanostructured phases [7].

O-I hybrid materials have been considered primarily for use as coatings in so far as they offer several advantages over ceramics and, in particular, the possibility of “curing” at low temperatures, thereby eliminating the need to carry out a final sintering operation at elevated temperatures [1,2,3,4]. Among these materials, there are the hybrid free radical-cationically photopolymerizable systems.

Several hybrid UV-curing systems using mixtures of acrylates and epoxides [8,9], vinyl ethers [10,11] or oxetanes [12,13] were recently developed. One of the main advantages of these systems is that the oxygen inhibition of free radical polymerization is greatly reduced, thus obtaining higher photopolymerization rates and conversions, as well as reduced costs by eliminating nitrogen blanketing normally used in radical photopolymerization processes. The UV-induced crosslinking reactions might be followed by hydrolysis-condensation (sol-gel process) of tetra-alkoxysilane and condensation with the alkoxysilane groups of the coupling agent: this leads to a silica nanophase strictly interconnected with the polymer matrix [14,15].

It is quite well established that O-I hybrids have excellent mechanical properties, particularly hardness and wear resistance, as well as an outstanding chemical stability towards solar radiation and atmospheric pollutants [16,17,18]. These characteristics are particularly attractive for high-performance coatings, *i.e.*, those employed for the protection of high value items of cultural heritage, historical buildings, monuments and wood artwork. The main modifications taking place in stone and wood exposed to outdoor elements arise from a combination of physical, biological and chemical phenomena, where water always plays a prominent role [19]. In order to preserve historical/artistic heritage from degrading phenomena, the use of protective coatings on affected areas and/or the consolidation and gluing of weakened parts [20,21,22,23] are often employed. The major essential properties required for protective/consolidating agents can be summarized as follows [24,25]: hydro-repellence, permeability to water vapor, reversibility or the possibility to re-treat the surface, durability, transparency and compatibility with the substrate and the capability to prevent the infiltration of contaminants or degrading agents from the atmosphere. The polymers normally used as protective/consolidating agents for stone structures and elements are usually formulations based on epoxies, acrylics and metacrylics, silicones, fluoroligomers and urethanes. To achieve a better resistance to weathering and to confer hydro-repellence, into the resin mixtures are often added specifically designed silanes and siloxanes containing acrylic functional groups [24,25]. The application of coatings, using brushes or spraying equipment, is achieved through their dispersion in low viscosity organic solutions or, if possible, in water. The use of organic solvents is frowned upon, due to their toxicity, which endangers the operator and pollutes the environment, owing to their fast rate of evaporation. Protective coatings based on aqueous dispersions are obviously preferred, since they are free from toxic hazard and can be, in addition, quite economical. On the other hand, they are pretty sensitive to humid environments, thus displaying poor durability resistance [24,25]. All coating systems commercially available for stone protection have a very limited capability to allow the migration of water vapor, this feature being, on the other hand, a fundamental requirement for these applications.

The use of O-I hybrids based on epoxy resins has been first suggested in the field of restoration/protection of masonry historical buildings by Cardiano *et al.* [26]. However, the poor resistance of the organic component to solar radiation was reported to limit its use in such applications. This phenomenon is explained by the evolution of ethanol in the sol-gel reactions, which could give rise to the formation of bubbles and/or plasticize the organic phase.

In the present paper, a novel procedure to obtain a UV-photopolymerizable acrylic-based hybrid formulation, usable as a protective coating of stone elements, was proposed. The method combines the sol-gel reaction, involving as the silica precursor tetraethoxysilane (TEOS), with a photopolymerization process. The latter technique is becoming increasingly important in the field of coatings [27,28]: it induces the polymer formation with a fast transformation of the liquid monomer into a solid film possessing tailored physical-chemical and mechanical properties. In addition, it is considered an environmental friendly technique, due to the solvent-free process, usually carried out at room temperature [27,28,29]. The properties and characteristics of an uncured and a dual-cured hybrid system were deeply analyzed and compared with those displayed by a control organic formulation.

## 2. Experimental Section

### 2.1. Materials

Trimethylolpropane trimethacrylate (TMPTMA) was chosen for its high reactivity and low viscosity (0.045 Pa·s at 25 °C). The product used was supplied by Cray Valley (Colombes, France). The content of resin was 85% in the total weight of the organic fraction for both mixtures produced, *i.e.*, control and hybrid systems.

A trimethoxypropyl silane methacrylate monomer, produced by Dow Corning (Midland, MI, USA) as Z6030, known as MEMO, was used as a precursor for the preparation of the O-I hybrid systems.

A vinyl terminated polydimethylsiloxane (VT PDMS), supplied by Aldrich (St. Louis, MO, USA), was also added to the acrylic mixture. The amounts of MEMO and VT PDMS were chosen in order to achieve about 3% of silica in the control formulation. The same amount was, then, used for the hybrid system.

Tetraethoxysilane (TEOS), supplied by Aldrich, was used as the alkoxide precursor for the hydrolysis and condensation reactions. The amount of TEOS used was determined producing different formulations in order to achieve a further 5% wt content of silica in the hybrid system.

3-Mercaptopropyltriethoxysilane (MPTS), supplied by Aldrich, was added to the siloxane-modified methacrylic resin system in order to reduce the effect of inhibition of oxygen toward radical photopolymerization. The functionalization of VT PDMS with MPTS was performed by mixing the two components at 100 °C in a 1:1 molar ration in the presence of 1% wt of diethylamine, again supplied by Aldrich.

**Scheme 1:**
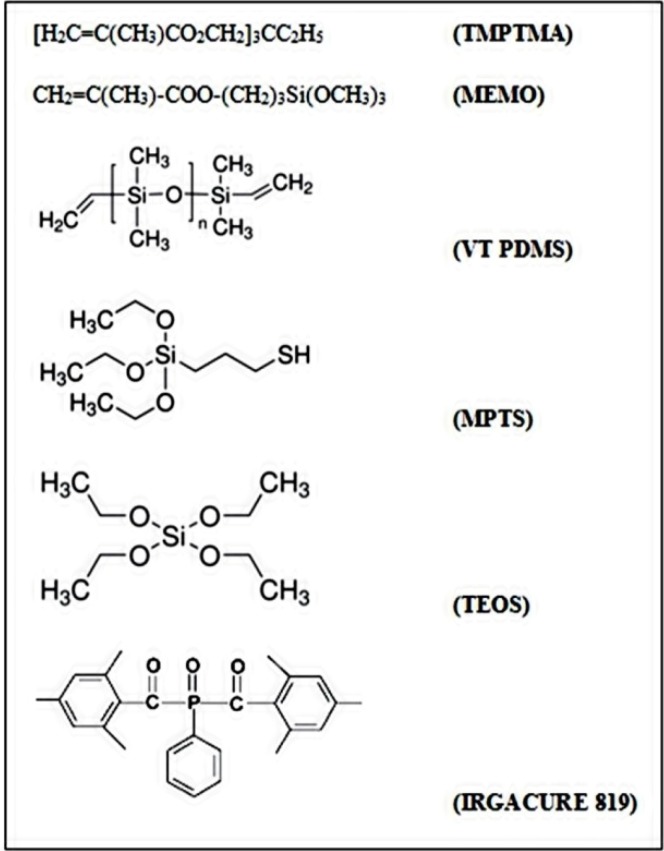
Chemical formula of each component used. TMPTMA, Trimethylolpropane trimethacrylate; MEMO; VT PDMS, vinyl terminated polydimethylsiloxane; MPTS, 3-Mercaptopropyltriethoxysilane; TEOS, tetraethoxysilane.

The photoinitiator, Irgacure 819, supplied by Ciba (Basel, Switzerland), was chosen for its broad absorption characteristics under UV region radiation. The structure of all the components used is shown in Scheme 1.

The rationale for the material selection is outlined next. The obtained co-continuous hybrid system is composed of nanosized silica domains (or very dense siloxane networks), whose presence is assured by the relative easiness with which hydrolysis and condensation reactions can be controlled by an alkoxide precursor, such as TEOS. MEMO was selected as the silane coupling agent to chemically link together the organic and inorganic components via the formation of suitably structured interphases, thus preventing the gross phase separation and the formation of particulate domains. The MPTS was selected, since it is able to counteract the inhibition effect carried out by oxygen towards the photopolymerization reactions [30]. The polydimethylsiloxane functionalized with MPTS (PDMS_m_) was added to the alkoxide precursor to obtain an organic-inorganic acrylic-silica network produced by a dual mechanism, *i.e.*, the condensation reaction of the hydrolyzed alkoxy groups and autocatalytic free radical polymerization, respectively. The presence of PDMS blocks in the organic phase gives rise to hydro-repellence characteristics in the coatings. Other advantages arise from the presence of a large quantity of SiOH groups within the inorganic phase, which will exert a strong affinity on the selected substrate (*i.e.*, stone). Moreover, the presence of co-continuous nanophases containing PDMS blocks dispersed in the very rigid silica domains are expected to confer to the coating good mechanical properties and the retention of these on aging, ensuring a high durability of the product. Finally, the inorganic domains are formed before the crosslinking reactions of the organic domains take place. As a consequence, the expected volumetric shrinkage, due to crosslinking reactions of methacrylic resin, cannot occur on a macroscale, due to the constraints imposed by the surrounding rigid silica domains. Accordingly, the porosity of the cured protective film is expected to take the form of a nanocapillary, which results from the inversion of the micellae present when adding the organic monomer to the inorganic precursor (hydrolyzed TEOS) [29]. This is a highly desirable feature, since it allows the migration of water vapor from the substrate (stone or wood) toward the outside.

#### Preparation of Hybrid System

The proposed polymerizable organic precursor consists of a mixture of VT PDMS functionalized with MPTS, as previously described [30], and methacrylic resin TMPTMA. The silane organofunctional MEMO was, then, added to the organic precursor based on PDMS_m_ and TMPTMA. The crosslinking reactions of the proposed formulation were induced by a standard photoinitiator for free radical polymerization, Irgacure 819. This system constitutes the control formulation (Ctrl Silane) used to analyze the polymerization kinetics. The exact procedure used for the preparation of the Ctrl Silane formulation is covered by a European patent [31].

For the synthesis of the hybrid system (indicated as Hybrid_85T_), MEMO and TEOS were first mixed at room temperature, using an excess of water, with a suitable amount of HCl, which is a well-known catalyst to promote the hydrolysis reactions. An appropriate amount of ethanol was also added to assist the solubilization of the alkoxysilane mixture in water. To this mixture was, then, added the organic fraction, previously photo-initiated. The obtained system was mixed and stirred under appropriate conditions. The complete procedure is, again, covered by a European patent [31]. The sol-gel reactions involved in the process are shown below:

**Step 1. Hydrolysis**


Si(OEt)_4_ + *n* H_2_O → (HO)*_n_*Si(OEt)_4-*n*_ + *n* EtOH (1)

Si(OEt)_4_ + *n* H_2_O → (HO)*_n_*Si(OEt)_4-*n*_ + *n* EtOH (2)

Oligomer[Si(OEt)_3_]*_x_* + *m* H_2_O → Oligomer(HO)*_m_* [Si(OEt)_3-*m*_]*_x_* + *m* EtOH (3)

**Step 2. Condensation**


≡SiOH + HOSi≡ → ≡Si-O-Si≡ + H_2_O (4)

≡SiOEt + HOSiO≡ → ≡Si-O-Si≡ + HOEt
(5)

The hydrolysis and condensation reactions produce the building blocks (inorganic nano-blocks), which can assemble together to produce co-continuous domains within the organic phase (organic nano-blocks), the latter represented in the above scheme by the Oligomer segment. Co-reactive silane coupling agents, containing alkoxysilane groups and functional groups that can react with the oligomer, are frequently used to assist the formation of co-continuous nano-phases.

### 2.2. Experimental Techniques

Different characterization techniques were used to analyze the liquid (un-reacted) and crosslinked hybrid system, comparing it with the control one.

Wide-angle X-ray diffraction of the powder, obtained starting from a liquid (uncured) hybrid system burned out in oven at 800 °C in air atmosphere, was collected on a PW 1729 Philips, using Cu Kα radiation in reflection mode (λ = 0.154 nm). The samples were step-scanned at room temperature from 2θ values of 10°–60°. This technique was used to initially identify the chemical nature of the inorganic particles formed during sol-gel reactions.

The size of the same particles formed during the sol-gel process, their distribution and stability in time, up to 12 months, starting from their preparation, in the liquid mixture was measured by dynamic light scattering (DLS Zetasizer).

The rheological characterization of both liquid formulations, *i.e.*, control and hybrid mixtures, was carried out in a strain controlled Rheometer (Ares Rheometric Scientific). The tests were performed with a plate and plate flow geometry (radius = 12.5 mm) in steady state mode and at room temperature (25 °C), using a shear rate ranging from 0.05 to 100 s^−1^. The rheological experiments were repeated at least three times to check the repeatability of results. The rheological tests were performed on both liquid systems after long storing times, *i.e.*, five months and one year, in order to analyze the stability of each formulation.

The solid residual of each liquid formulation were determined by thermogravimetric analysis (TGA/DSC1 Star and System, METTLER Toledo, Zürich, Switzerland). To this aim, the samples were heated from room temperature up to 800 °C at 10 °C/min in nitrogen atmosphere. Three replicas were performed on each system.

Fourier transform infrared (FTIR-6300 Jasco, Easton, MD, USA) and Raman/FT-6000 (Jasco) spectrometers were used to monitor the consumption of the reactive species in the liquid mixtures and, in turn, to analyze the reaction mechanism. Preliminarily, FTIR and Raman analyses were performed on a thin layer of uncured mixtures, using a KBr support. The same sample was, then, irradiated every 10 s up to complete polymerization (5 h) with an irradiation intensity of 3.7 µW/mm^2^. Each sample was, finally, heated in an oven at 140 °C for 1 hour. After each polymerization step, the sample on the support was placed in the FTIR and/or Raman instrument, in order to determine its characteristic peaks.

Both formulations were, then, coated on a glass substrate and photo-cured by using a medium pressure Hg UV lamp (UV HG 200 ULTRA, Ultra Electronics, London, UK), with a radiation intensity on the surface of the sample of 9.60 µW/mm^2^ at 365 nm working in air atmosphere. The maximum time used for the complete curing of the films was five hours, as experienced in previous work [22,30]. A cured film possessing a thickness of about 150 µm was obtained. After a 5 h photopolymerization step, the samples were moved to an oven and kept at 140 °C for one hour, in order to complete the radical crosslinking reaction with a thermal stage. This procedure, consisting of a first step of photopolymerization followed by a second step of thermal cure, is known in the literature as “dual-curing”. In the dual-curing process, the sol-gel reactions of inorganic precursors are combined with the polymerization of mono- or multi-functional monomers. The formation of the inorganic moieties is due to the polycondensation of the metal alkoxides, which takes place after their hydrolysis, while the organic matrix originates from the polymerization of the organic monomers. The use of a suitable coupling agent, containing both a polymerizable functional group and an alkoxide group, provides covalent bonding between the organic and inorganic phases. Therefore, a network with a strict and strong interconnection between the inorganic domains and the polymer can be formed [32]. The dual-curing process can be classified as a bottom-up method for making nanostructured materials and prevents macroscopic phase separation: the control of the process conditions and of the interactions between the components rules the size of the inorganic clusters, which can vary from nano- to sub-micron size. On the contrary, top-down processes based on the dispersion of pre-formed nanoparticles often cannot avoid agglomeration [33].

Transmittance spectra of coatings, obtained by dual-curing both formulations, were recorded at normal light incidence in the spectral range of a wavelength 300–1100 nm with a Varian Cary 50 SCAN UV-Vis Spectrophotometer. The thickness of the tested samples was 150 µm.

The morphology of dual-cured hybrid film, obtained as described above, was characterized by Scanning electron microscopy (SEM) using a Jeol JSM-6550F.

The thermal stability of control and hybrid films was measured by thermogravimetric analysis (TGA/DSC1 Star and System, METTLER Toledo). To this aim, the samples were heated from room temperature up to 800 °C at 10 °C/min in nitrogen atmosphere. Three replicas were performed on each system.

The glass transition temperature (*T*_g_) of dual-cured specimens was measured using an Ares Rheometric Scientific Dynamical Mechanical Thermo-Analyzer (DMTA, Rheometric Scientific Inc. Piscataway, NJ, USA). Samples of both systems (about 3.5 mm × 10 mm × 3 mm in size) were subjected to an oscillation in torsion mode of a constant amplitude (10%) and frequency (1 Hz) during their heating in air from room temperature up to 150 °C, using a heating rate of 5 °C/min.

*T*_g_ measurements of the same dual-cured samples were also performed by using other Thermal Analysis Instruments (Mettler Toledo DSC1 StareSystem and Perkin Elmer Thermomechanical Analyzer TMA 7), heating 10–20 mg of each sample from 20° C to 250 °C at 10 °C/min; both measurements were performed in air atmosphere. For comparison purposes, three specimens for each formulation were analyzed and the results averaged.

## 3. Results and Discussion

**Figure 1:**
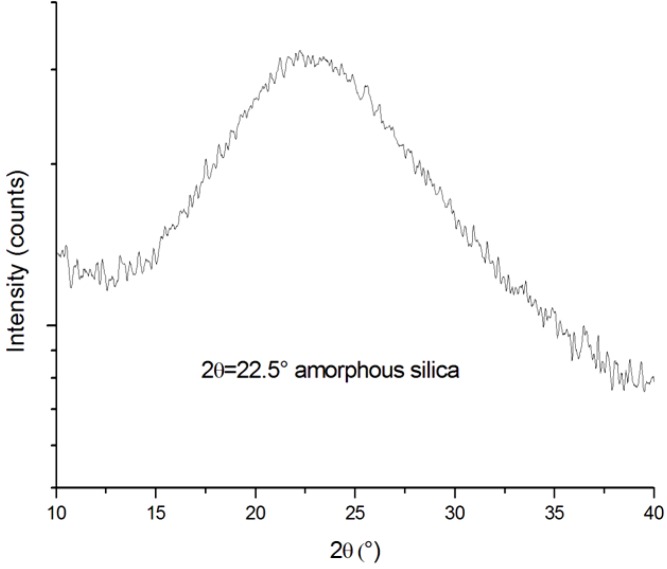
XRD spectrogram of amorphous silica obtained after burning out uncured Hybrid_85T_ formulation.

First of all, the nature of the particles formed through hydrolysis and condensation reactions in the hybrid mixture was investigated. To this aim, the solid residual of the liquid Hybrid_85T_ formulation, burned out at 800 °C, was analyzed by X-ray powder diffraction (XRD) analysis. The XRD pattern of the resulting powder, reported in Figure 1, reveals that the hybrid mixture contains amorphous silica powders.

The XRD spectrum, in fact, presents a wide peak at about 2θ = 22.5°, which corresponds to the peak representative of the amorphous silica, as reported in the literature [34].

**Figure 2:**
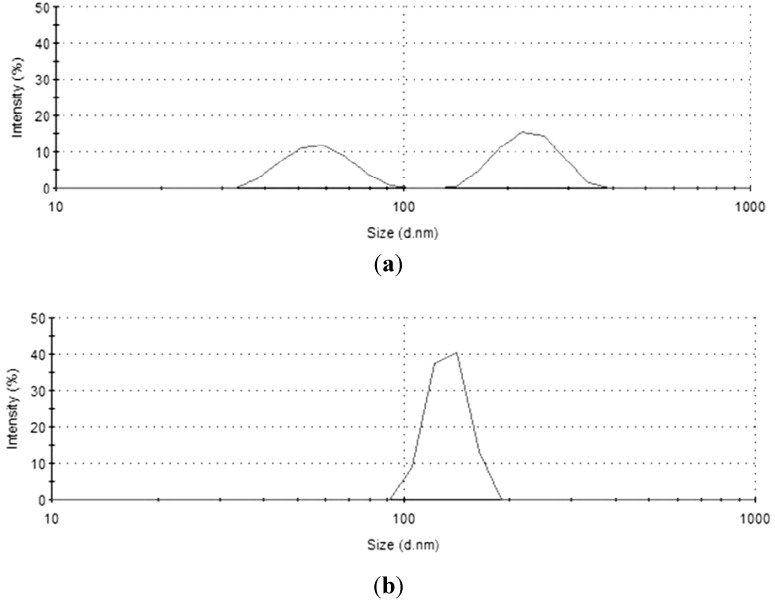
Particle size distribution calculated through dynamic light scattering (DLS) analysis for uncured Hybrid_85T_ formulation (**a**) after one day and (**b**) after five months.

The particle size distribution of the hybrid mixture measured by dynamic light scattering (DLS), after one day and five months from the preparation, is reported in Figure 2.

The particle size distribution measured one day after the preparation appears bimodal; in particular, about 55% of the particles have a diameter around 232 nm, and the remaining 45% of the particles have a diameter of about 57 nm. After five months, the size distribution of the same mixture becomes unimodal, *i.e.*, all the particles have a homogeneous diameter of about 134 nm. This result suggests that the sol-gel process is able to form silica nanometric particles within a polymeric (acrylic-based) matrix, confirming the efficiency of the method used. However, these particles are not stable in time: after a long time from their preparation, their dimensions become uniform, but still with a nanometric size. This variation of the distribution of the dimensions of the silica particle could be attributed to the proceeding of the sol-gel reactions during the first five months from the preparation of the hybrid mixtures. As a consequence of the proceeding of hydrolysis and condensations reactions occurring during this additional time, the average diameter of all silica particles produced was found to be reduced. However, tests performed after one year from the preparation (not shown in the present paper) confirmed that the particles maintain the same dimension for very long times. This result confirms that the sol-gel reactions were terminated after five months, and starting from this period, the average dimension of the silica powder remained almost unchanged; consequently, the mixture was found to be stable during the subsequent storing time, up to one year. The DLS analysis performed on the organic mixture after the same time from the preparation showed, as expected, shows that this mixture does not contain nanometric particles, not at the beginning, neither after five months from the preparation. DLS results obtained on organic and hybrid mixtures are summarized in Table 1.

**Table 1:** Results from DLS analysis performed on uncured Ctrl Silane and Hybrid_85T_ formulations.

Mixture	After 1 day		After 5 months	
	Intensity (%)	Particles diameter size (nm)	Intensity (%)	Particles diameter size (nm)
Ctrl Silane	100	>6,000	100	>6,000
Hybrid_85T_	54.8	231.8	100	134.3
	45.2	57.4		

**Figure 3:**
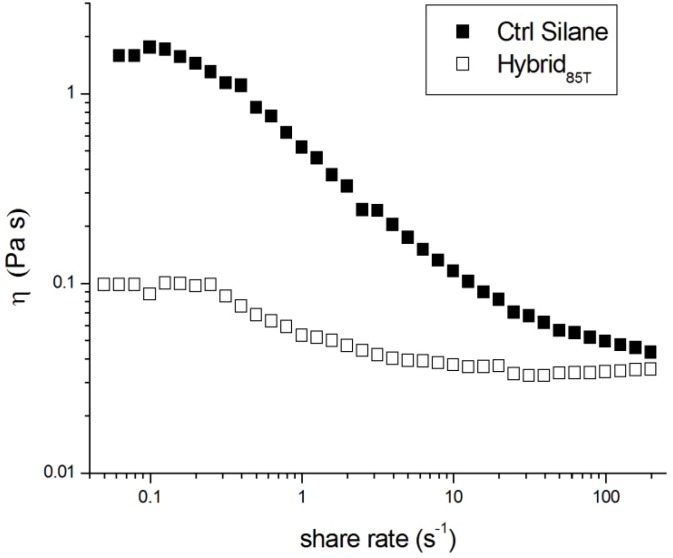
Rheological curves of uncured Ctrl Silane and Hybrid_85T_ formulations.

The rheological behavior of both acrylic-based formulations was, then, investigated as a function of the shear rate at room temperature, in order to analyze the fluidity of both mixtures, which is particularly important for possible applications. A low viscosity is, in fact, always required to easily apply polymeric photopolymerizable formulations as coatings for different substrates, by using spray or brush medium. As an example, an adequate value of viscosity for the application of a monomeric formulation as a protective coating for porous stone elements is about 0.3–0.5 Pa·s [35]. On the other hand, the knowledge of the viscosity of a coating as a function of shear rate is necessary in order to establish the range of shear rate suitable to obtain the best fluidity of the mixture for the specific application. The rheological curves for the uncured systems analyzed are reported in Figure 3.

For both formulations, as expected, a pseudo-plastic behavior, with a viscosity decreasing with shear rate, was observed. A pseudo-plastic behavior is, in fact, very common for polymeric resins of medium viscosity [35]. However, in the case of the hybrid system, this behavior is less evident, *i.e.*, the pseudo-plastic portion of the curve is limited to shear rates varying between 0.3 s^−1^ to 5 s^−1^, and the corresponding measured viscosity changes from 0.085 Pa·s to 0.039 Pa·s. For lower and higher share rates, up to 200 s^−1^, the viscosity of the Hybrid_85T_ system remains almost constant. The observation of a slightly pseudo-plastic rheological behavior, limited at a short intermediate shear rate range, for the hybrid system suggests the absence of any silica particle settling, evidencing, in turn, the efficiency of the fixed test conditions (temperature, pH, time and rate of mixing) for the sol-gel method to produce separated silica nanoparticles in a liquid organic matrix. Moreover, in all the ranges of shear rates investigated, the viscosity of the hybrid mixture was found to be much lower than that of the Ctrl Silane system, of about one order of magnitude. This result, attributed to the presence of TEOS acting as plasticizer for the acrylic mixture, could represent an important advantage in the application stage, in particular when using a spray technique.

The rheological behavior of both formulations was also monitored as a function of storing time, *i.e.*, at five and 12 months from their preparation. The viscosity curves obtained for both systems were always equal to those reported in Figure 3, evidencing the stability of the formulations, even after one year from their production. This result, confirming that previously found with DLS analysis, is a further indirect evidence of the capability of the preparation method used to produce an interconnected stable organic-inorganic structure, with no separation phase.

Both liquid mixtures were, then, analyzed by thermo-gravimetric analyzer, TGA, to quantify the silica content, in comparison with the solid residue calculated on any single component. For comparison purposes, in fact, the silane organofunctional, MEMO, the methacrylic resin, TMPTMA, and the siloxane monomer functionalized with the thiol monomer, MPTS, *i.e.*, PDMS_m_, were also analyzed with the thermogravimetric technique. The solid residue calculated only on both mixtures is summarized in Table 2.

**Table 2:** Solid residue calculated from thermogravimetric analysis (TGA) analysis performed on uncured Ctrl Silane and Hybrid_85T_ formulations.

Sample	Char (%)	Std. Dev. (%)
Ctrl Silane	3.7	5
Hybrid_85T_	8.8	3

The Ctrl Silane mixture shows a total weight loss of about 96.3%. The Si-O groups initially present in methacrylic resin, PDMS_m_ and MEMO, therefore, were transformed in silica aggregate (3.7 wt %) in control formulation. The hybrid formulation, on the other hand, shows a final residual content of about 9 wt %. The difference of this residual with that measured on the Ctrl Silane, *i.e.*, about 5%, corresponds to the silica formed as a consequence of the sol-gel reactions brought about by TEOS. The TEOS amount used was, in fact, optimized in order to achieve an additional content of nano-structured silica of 5 wt % in the hybrid system before the crosslinking of the organic phase.

**Figure 4:**
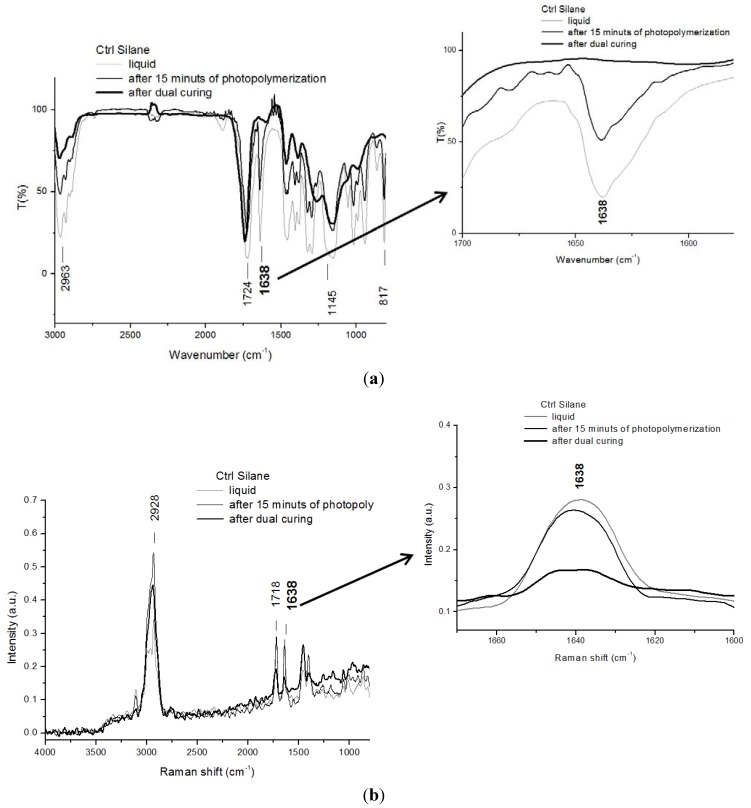
(**a**) Fourier transform infrared (FTIR) spectra of Ctrl Silane formulation at different stages of curing. In the frame on the right, there is enlargement of the peak at 1638 cm^−1^; and (**b**) Raman spectra of Ctrl Silane formulation at different stages of curing. In the frame on the right, there is enlargement of the peak at 1638 cm^−1^.

FTIR and Raman analyses were performed on the two systems before and after the photopolymerization process at different times, ranging from 0 to 18,000 s. The same analyses were also performed after the dual-curing treatment. Results of FTIR and Raman spectroscopy, performed on the control system before and after 15 min of photopolymerization and after the dual-curing treatment, are reported in Figure 4a,b, respectively.

From the analysis of all FTIR spectra reported in Figure 4a (on the left side), the presence of the peaks relative to the stretching asymmetric C–H bonds at 2963 cm^−1^, attributed to the chemical structures of the TMPTMA acrylic resin, the organofunctional silane MEMO and the thiol MPTS, the symmetric stretching C=O bond at 1724 cm^−1^ and the stretching C=C bond at 1638 cm^−1^, both relative to TMPTMA and MEMO resins, can be noticed. Si–O–Si linkages can be also observed in all the spectra at 1145 cm^−1^, due to the presence of MEMO and MPTS monomers in the mixture. At 817 cm^−1^, the presence of the stretching Si–C bond can be noticed, due to the PDMS monomer. The spectra of the Ctrl mixture shows a progressive decreasing of the intensity of the more significant peak at 1638 cm^−1^, as a function of the time of UV lamp irradiation. The reduction of the height of this peak is particularly evident in the spectrum obtained on the sample after 5 h of UV irradiation (not shown) and after the subsequent heating in the oven at 140 °C, *i.e.*, after the dual-curing treatment. These results confirm that the radical reaction took place at the test conditions analyzed, though it was not complete, even after the dual-curing treatment, since the peak at 1638 cm^−1^ is still present, as better evidenced in the enlargement shown on the right side of Figure 4a.

In Figure 4b, the Raman spectra of the control system, before and after 15 min of UV lamp exposure and dual-curing treatment, appear comparable to those obtained by FTIR analysis, since the presence of the same peaks observed in Figure 4a is noticed. The stretching asymmetric C–H, present at 2963 cm^−1^ in Figure 4a and relative to the chemical structure of TMPTMA acrylic resin, is now shifted to 2928 cm^−1^; the symmetric stretching C=O bond present at 1724 cm^−1^ in the FTIR spectra is now shifted to 1718 cm^−1^; the stretching C=C bond relative to TMPTMA and MEMO monomers is still present, but at 1638 cm^−1^. The identification of the peaks related to Si–O–Si and Si–C linkages was more difficult in Raman spectra. There are also two additional peaks visible, at 1463 cm^−1^ and 1395 cm^−1^, again attributed to C–H bonds. Additionally, in this case, the characteristic peak of the double bound C=C decreases by increasing the time of UV exposure and after the heating treatment. This result confirms that the radical reaction took place in the test conditions analyzed. The reaction was not complete, even after the dual-curing treatment, since the peak at 1638 cm^−1^ is still present, as, again, clearly shown in the enlargement on the right side of Figure 4b.

**Figure 5:**
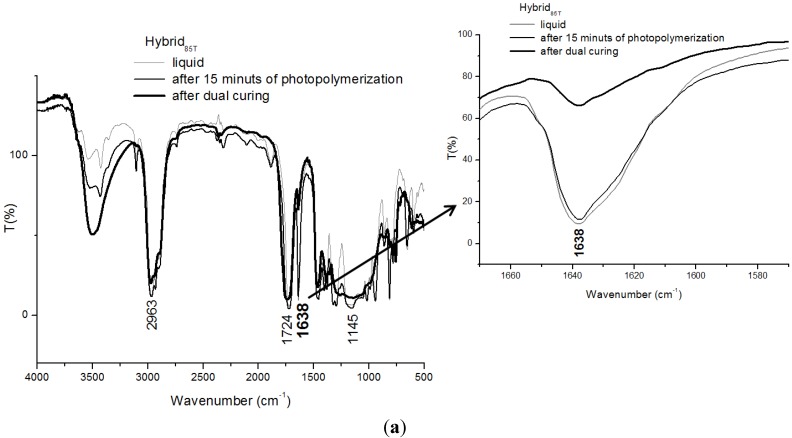

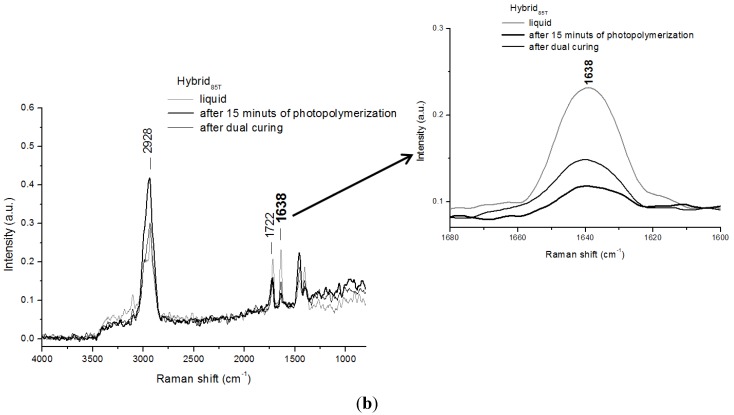
(**a**) FTIR spectra of Hybrid_85T_ formulation at different stages of curing. In the frame on the right, there is enlargement of the peak at 1638 cm^−1^; and (**b**) Raman spectra of Hybrid_85T_ formulation at different stages of curing. In the frame on the right, there is enlargement of the peak at 1638 cm^−1^.

FTIR and Raman spectra of hybrid formulation, before, after 15 min of UV lamp irradiation and after dual-curing treatment, are reported in Figure 5a,b, respectively.

The presence of the peaks at 2963 cm^−1^, 1724 cm^−1^ and 1638 cm^−1^, relative to the organic monomers, can be still noticed in all the spectra reported in Figure 5a. In addition, the Si–O–Si linkages and Si–C bond, at 1145 cm^−1^, are still present in the FTIR curve of the liquid hybrid mixture. However, these latter ones disappear in the spectrum of the same system after 15 min of the photopolymerization process and dual-curing treatment. This result suggests that Si–O–Si and Si–C bonds have been opened to allow the creation of inorganic silica domains, as a consequence of the photopolymerization reaction. On the other hand, silica nanostructured domains were also obtained by the elimination of water from the SiOOH groups, as a consequence of the condensation reactions, occurring during the sol-gel process [14].

The spectra of the hybrid mixture after dual-curing treatment shows a greater decrease of the intensity of the more significant peak at 1638 cm^−1^ in comparison to that of the organic system, as evidenced in the enlargement of the peak, displayed on the right side in Figure 5a. This result suggests that the hybrid mixture is able to react with a higher rate with respect to the organic formulation.

From the analysis of the Raman spectra of the hybrid system, reported in Figure 5b, it can be noticed that, again, it is difficult to identify the peaks related to the Si–O–Si linkages and Si–C bond in the spectra. It can be also noticed that the peaks related to the organic fraction, present in the spectra of the Ctrl system, are still evident in the spectra of the hybrid formulation, before and after the photopolymerization reaction. In particular, the stretching asymmetric C–H bond can be identified at 2928 cm^−1^, at 1463 cm^−1^ and 1395 cm^−1^ and the symmetric stretching C=O at 1722 cm^−1^ and the stretching C=C bond at 1638 cm^−1^. Again, the characteristic peak of the double bound C=C, enlarged in the right side of Figure 5b, appreciably decreases after the photopolymerization reaction and the dual-curing treatment.

Starting from FTIR spectra of both the mixtures, before and after the photopolymerization reaction, at different times of UV lamp exposition and thermal treatment, the maximum extent of reaction (α) for both systems was determined as follows [8]:

(6) $$\alpha =\frac{{\left(A_{C=C}\right)}_{ti}-{\left(A_{C=C}\right)}_{t0}}{A_{C=O}}$$

where (*A*_C=C_)*_ti_* is the absorbance related to the C=C bond at the time, *t_i_*; (*A*_C=C_)*_t_*_0_ is the absorbance related to the C=C bond the initial time, *t*_0_; and *A*_C=O_ is the absorbance of the invariant C=O bond.

The maximum extent of reaction α for the two formulations analyzed, obtained starting from FTIR spectra, was α_FTIR_ = 70% for the hybrid system and 25% for the organic one.

**Figure 6:**
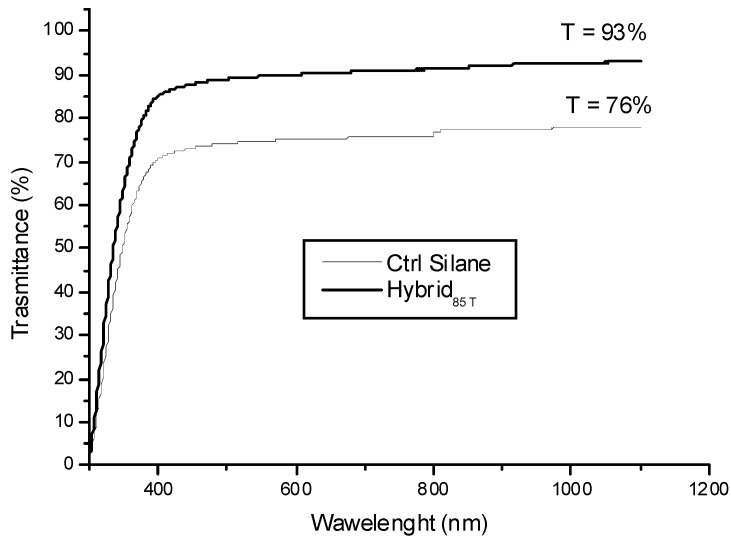
Light transmittance spectra of dual-cured Ctrl Silane and Hybrid_85T_ systems.

The optical transparency of the coatings, obtained by dual-curing both formulations, was determined by light transmittance measurements, over a range of 300 to 1200 nm. The comparison of light transmittance recorded for control and hybrid coatings is reported in Figure 6.

It is noticed that the light transmittance of siloxane-modified acrylic resin and hybrid coatings are about 76% and 93%, respectively. The noticeable increase of the optical transparency of the hybrid coating confirms that the rigid co-continuous silica domains have been homogeneously dispersed in the polymer matrix. In particular, this result suggests that the siloxane nanostructured domains (*i.e.*, primary particles with a diameter in the region of 0.5–0.9 nm and consisting of a few hydrolyzed and condensed TEOS and MEMO species) do not segregate in coarse domains (*i.e.*, larger than 450 nm). Transparency is regarded as evidence of a uniform distribution of the silica phase generated within the polymeric network and of an organic-inorganic phase separation on a scale significantly smaller than 400 nm [32]. The dual-curing process is confirmed to be a successful strategy for the *in situ* generation of inorganic domains starting from a liquid inorganic precursor. The presence of a coupling agent, which can interact with the polymeric growing chain from one side and with the inorganic network forming *in situ* on the other side, allows one to control the dimension of the inorganic clusters: nanometric size domains were, thus, obtained, well dispersed within the polymeric matrix. In particular, the hydrolysis and condensation reactions of the alkoxysilanes, MEMO and TEOS, are supposed to continue in an oven at 140 °C. These reactions can occur inside the polymer network, which has a flexible structure at the temperature set for the process, *i.e.*, above the *T*_g_ of the organic matrix. In these conditions, a good mobility of the system is assured, allowing the condensation reactions to carry on. The final result is an optically transparent hybrid material, the latter being a very important characteristic required for any film to be used as a protective coating for different substrates.

**Figure 7:**
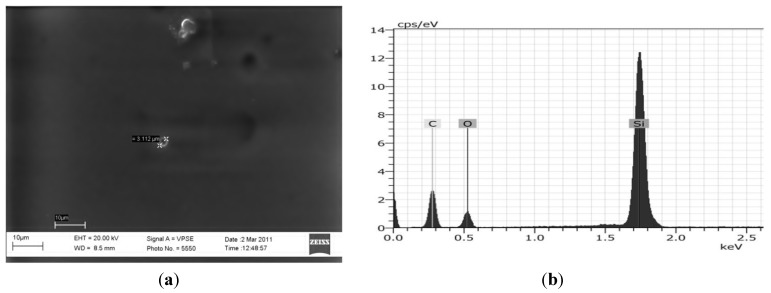
(**a**) Scanning electron microscopy (SEM) image of dual-cured Hybrid_85T_ applied on glass substrate; and (**b**) Energy-Dispersive X-ray spectroscopy (EDS) analysis of the same system.

Even if the hybrid films were transparent to the naked eye, as also confirmed by transmittance measurements, a microscopic phase separation was observed at the nanometric scale by SEM analysis, as reported in Figure 7.

The SEM image of the hybrid coating applied on glass substrate with the correspondent EDS analysis, reported in Figure 7, evidences, in fact, not only the presence of co-continuous silica domains, but also the presence of two isolated micrometric clusters, with an average size lower than 3 µm. Through EDS analysis, whose results are shown in Figure 7 (right side), it was confirmed that the aggregates are made of silica, since the Si signal is predominant. Similar results were also observed with other UV photopolymerizable polymer-silica hybrid composites: also in those cases, in fact, TEM micrographs revealed the presence of round micrometric particles made of silica, even if the dual-cured polymers produced were crack-free and transparent to visible light [32].

Apart from these two clusters, the hybrid coating displays the typical features of organic-inorganic hybrids, which consist of diffuse silica domains dispersed within an organic matrix. The micrograph reported in Figure 7 shows, in particular, that the organic and inorganic phases are strictly interconnected, with almost no major macroscopic phase separation that might have occurred during the dual-curing process. The silica domains, generated by the sol-gel process, are embedded in the polymeric matrix, producing strong interactions between the organic and inorganic network, attributed to the presence of MEMO as the coupling agent. By completely avoiding macroscopic phase separation, it would be possible to obtain a perfect hybrid material in the nanoscale range. To this aim, a modification of the test conditions employed during the sol-gel reactions, or of the composition of the hybrid formulation, is currently under investigation. The reduction of the dimensions of all the silica particles in the hybrid coating could be, in fact, necessary to further improve the transparency of the coating and to achieve superior surface properties.

**Figure 8:**
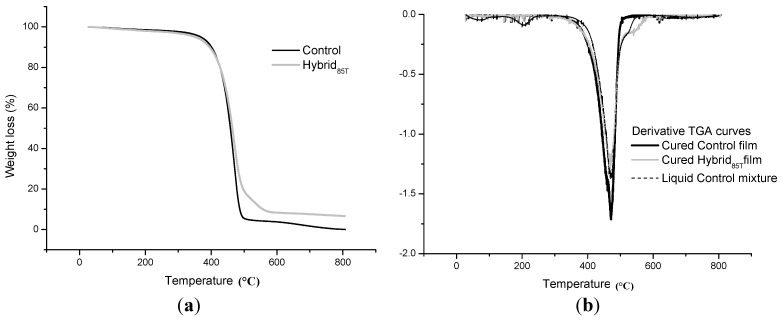
(**a**) TGA curves of dual-cured Ctrl Silane and Hybrid_85T_ systems; and (**b**) derivative TGA curves of dual-cured Ctrl Silane and Hybrid_85T_ systems.

The comparisons between the TGA curves of control and hybrid dual-cured films and of the relative derivative curves are shown in Figure 8a and Figure 8b, respectively. For comparison purposes, in Figure 8b, the derivative TGA curve for the uncured Hybrid_85T_ system is also reported.

The TGA curves found for both dual-cured systems are very similar, at least up to the decomposition onset. The decomposition peak temperature of the hybrid film (centered at 468 °C) is, in fact, only a few degrees lower than that of the control system (471 °C). This first step is attributed to the complete disappearance of organic components, irrespective of the system analyzed.

On the other hand, a second smaller peak around 550 °C in the derivative curve of the hybrid film is clearly evident. The same peak is never present in TGA analyses performed on the dual-cured Ctrl Silane system. In Figure 8b, the same peak observed for the dual-cured Hybrid_85T_ system in the derivative thermogravimetric (DTG) curve is found also for the uncured hybrid system, even if it is slightly anticipated (around 530 °C) and less sharp. The observed phenomenon, manifested by the peak in DTG curves for the dual-cured and uncured Hybrid_85T_ systems, might have been caused by the elimination of water from the SiOOH groups, transformed in SiO_2_, occurring at a high temperature (about 550 °C) after the complete degradation of the organic components. This hypothesis is supported by the results from FTIR and Raman analyses, which evidenced the presence of the characteristic peak of the Si–O–Si linkages and Si–C bond, at 1145 cm^−1^, only in the spectra of the liquid hybrid mixture. These latter peaks disappear in the spectrum of the same system after 15 min of photopolymerization processing and dual-curing treatment, confirming that that TEOS was not completely hydrolyzed during the preparation of the hybrid system, and consequently, the Si–O–Si and Si–C bonds have been opened to allow the creation of inorganic silica domains, as a consequence of the photopolymerization reaction. With increasing the temperature, in fact, the formation of the inorganic network proceeded continuously [36]. Furthermore, a solid residue of about 6.7% and 0.05% was found for the control and hybrid dual-cured films, respectively. By comparing these values with those obtained on the same systems in the liquid state, reported in Table 2, it is evident that the solid residues of the cured systems are lower than those of the liquid one. The photopolymerization process of the silane components present in the organic mixture avoided the formation of silica in the cured Ctrl Silane system, exhibiting, in fact, an almost null solid residue. In the case of the Hybrid_85T_ liquid mixture, about 5 wt % of silica on a total of around 9% was formed as a consequence of the hydrolysis and condensation reactions of TEOS, as previously explained. The higher experimental residue (6.7%) than the theoretical value (5%) obtained in the case of the cured hybrid film is probably due to the trapping of the polymer moiety in the silica network. The black color of the solid residue left after the TGA runs also provides evidence that a small percentage of organic moiety has been trapped in the silica matrix [37].

Finally, the glass transition temperature of the samples 3.5 mm × 10 mm × 3 mm in size, photocured for 5 h and post-cured in an oven for 1 h at 140 °C, was measured using DMTA, scanning calorimetry (DSC) and thermo mechanical analysis (TMA) dynamic tests. The values obtained with the different techniques are summarized in Table 3.

**Table 3:** *T*_g_ values measured on dual-cured Ctrl Silane and Hybrid_85T_ systems using different techniques. DSC, TMA, DMTA, dynamical mechanical thermo-analysis.

Sample	*T*_g_ (°C) by DSC analysis	*T*_g_ (°C) by TMA analysis	*T*_g_ (°C) by DMTA analysis
Ctrl Silane	46 ± 1	48 ± 2	53 ± 3
Hybrid_85T_	62 ± 2	63 ± 2	70 ± 4

By comparing the *T*_g_ values measured on the two dual-cured systems, it is clear that the hybrid system possesses a *T*_g_ appreciably higher (about 16 °C) than that calculated on the control system, irrespective of the technique used to measure it. As expected, the *T*_g_ calculated from the maximum of the loss tangent (tan δ) in DMTA curves is always substantially higher (7 °C or more) than those obtained from calorimetric experiments, *i.e.*, DSC and TMA. The observed results are, in fact, in line with those reported in the literature for different systems, such as for epoxy hybrid systems [3], attributed to a frequency effect. On the contrary, the values of *T*_g_ obtained from the other two techniques are very similar.

The great enhancement of the glass transition temperature found for Hybrid_85T_ is explained with the formation of co-continuous nanometric silica domains within the hybrid system: these inorganic domains are, in fact, responsible for the restriction of the chain mobility of the organic phase. Similar results were also observed with other polymer-silica hybrid composites [38,39]. Once TEOS is added and the condensation reaction occurred, in fact, the nanometric inorganic silica restricts the segmental motion of the polymeric chains, inducing an increase in *T*_g_ values, as observed in previous investigations on dual-cured hybrid materials [40,41,42,43]. In all these studies, the presence of nanoscale inorganic domains within the polymeric network and the condensation of alkoxy groups of TEOS and MEMO is overall effective in inducing an increase of crosslinking density. On the other hand, a *T*_g_ depression was observed for hybrid systems containing low silica contents, attributed to the plasticizing effects [44,45,46].

Then, the results obtained in the present study on the methacrylic-siloxane photopolymerizable hybrid system, in terms of a large improvement of the *T*_g_ of the organic mixture, represent an important advantage of this novel system, which further confirms the efficiency of the innovative method for the synthesis and the curing of the O-I hybrid coating. Furthermore, for the possible use of this system as a protective coating for different substrates, a high *T*_g_ could be very useful, not only to guarantee the perfect adhesion of the film on the substrates, but also to maintain its mechanical performance, even if the film is working under severe conditions. It is, in fact, well known that the absorption of water causes a *T*_g_ reduction of the coating, related to the plasticization effect of the water [3]. To this aim, the possibility to produce a coating whose *T*_g_ is appreciably higher than room temperature is an important advantage for the effective use of the novel system as a protective coating. Even if it will absorb water during outdoor exposure, in fact, its *T*_g_ will remain higher than room (environmental) temperature, avoiding any undesirable reduction of adhesion strength to the substrate and of the mechanical properties.

## 4. Conclusions

A photopolymerizable microgel modified UV-cured acrylic-silica hybrid formulation, for possible use as a protective coating of stone substrates, was developed and analyzed. The first part of the research dealt with the selection and optimization of suitable systems through appropriate chemical modifications, in such a way to ensure that crosslinking reactions of the organic and inorganic phases can be carried out at room temperature in the presence of UV radiation and take place in two consecutive steps.

The results of the experimental study allow us to conclude that co-continuous nano-sized silica domains strictly interconnected with the methacrylic-siloxane matrix were achieved in the hybrid system. The main advantages of the latter system can be summarized in a higher transparency and glass transition temperature in comparison to the Ctrl Silane system, even if its thermal degradation performance remains unmodified. The uncured hybrid system, moreover, possesses a long storing time.

Further studies are currently in progress to analyze the mechanical and surface properties of the hybrid system, in isolation or applied to a stone substrate.
